# Supplementary material for: Side effects may include: Consequence neglect in generating solutions
Source: PLoS One. 2025 Apr 30;20(4):e0322149. doi: 10.1371/journal.pone.0322149 (PMC12043123; doi:10.1371/journal.pone.0322149)
Supplement: Attachment — Submitted filename: Response to Reviewers.pdf [file pone.0322149.s002.pdf]

Dear Dr. Otterbring,

Thank you for your invitation to revise and resubmit our manuscript "Side Effects May Include: Consequence Neglect in Generating Solutions" for PLoS One. The reviews were excellent and helpful. We were able to improve the manuscript considerably based on the feedback. In particular:

1) We clarified our analyses, and ran several new analyses - while none of the results qualitatively changed, we feel the paper is improved by having improved our statistical treatment of our data.

2) We have expanded our literature review to incorporate a broader scope. We now reference a third more articles than in our previous version of the manuscript, and we believe our paper is much better situated in the extent literature as a result.

3) We have clarified the definitions of our terms and more clearly articulated the distinctions and relationships between our experimental constructs.

We also made a number of smaller edits in response to specific reviewer comments, caught various minor issues, and updated our text for clarity and flow. Below, we detail our response to each comment from you and the reviewers. The Reviewer or AE comment is in italics, and our response is in plain font. Thank you for the thoughtful and constructive feedback on our paper. We believe the manuscript is greatly improved, and hope you agree that it is now suitable for publication.

Sincerely,

Chris Rodriguez and Danny Oppenheimer

EDITOR COMMENTS

Both the reviewers raise many valid concerns related to your conceptualization, construct definitions, treatment of confounding factors, transparency aspects, and analytic decisions. Based on my own reading of the manuscript, I am willing to move your manuscript into another round of reviews. Given the magnitude of some of the raised issues, this will be a major revision, albeit a revision with a relatively clear path toward

publication as long as you meticulously address all the valuable feedback points from the reviewers in your revision.

We have devoted considerable time and attention dealing with these concerns; we describe the changes we made in our detailed response to reviewers below. We agree that the reviewers gave us very clear feedback, highlighting a clear path toward improvement and (hopefully) publication. We have attempted to be very responsive to reviewer concerns, and hope you agree that the manuscript is much improved as a result.

*One reviewer requests additional data in at least one new study. While I am convinced that such a study will bolster the quality and contribution of your empirical package, I am agnostic as to whether you *must* include more data as part of this submission. Thus, if you believe that you can compellingly address some of the issues identified through your existing data, you might be able to convince this reviewer that your work is still worthy of publication even without added empirical evidence. That said, the decision whether or not to collect additional data is ultimately your call.*

We appreciate your flexibility on the topic of whether we run an additional study. Ultimately, we decided against doing so, because we believe we were able to address the reviewers' concerns without new data. Some of the reviewer's concerns were due to our lack of clarity in the previous submission that particular issues had already been addressed. For example, the issue of participant fatigue was explicitly controlled for in Study 3, but we had not made that clear. That, along with several other miscommunications have hopefully been resolved, and have obviated the need for an additional study.

Reviewer #1:

While the concept may not be entirely new (considering the closely related/similar concepts of focalism and opportunity cost neglect mentioned by the authors), the current research nicely extends previous findings to the context of policies in various domains (e.g., education, organizational, urban planning, public health).
The manuscript is well-written, has a clear structure, and displays transparency. It is based on studies that build on each other in a meaningful way and address several limitations (specifically, Study 3), further demonstrating the robustness of the findings.

Overall, there are only a few minor aspects the authors could address for clarification and to further improve their manuscript. These issues will be briefly outlined below (in no particular order).

We thank the reviewer for the kind words, and for recognizing the value and contribution of our research. :)

#1. Literature/References
The authors could consider introducing additional relevant references, as the current literature base is relatively scarce (i.e., 13 references overall, including introduction/theory and discussion). This does not mean that the authors should add sources just to increase the number of references, but they may consider slightly extending their theoretical reasoning (e.g., from the literature on heuristics and biases) and/or discussion in the form of a few additional paragraphs and/or relevant sources.

Thank you for this feedback. In our original manuscript we had aimed to be as concise as possible, to make this paper accessible to non-academic readers (the general public and practitioners) but we acknowledge that we got the balance between concision and appropriately situating the manuscript in the extant literature wrong. We have added more discussion (and citations) to address that. In particular, we now discuss the literature on "predictable surprises", the literature on preference reversals from focalism interventions, and the heuristics and biases literature more generally. We appreciate you highlighting the need for additional literature review.

#2. Effect Sizes
In addition to t- and p-values, the author should consider reporting effect sizes for their studies, as these are currently not included in the manuscript.

Yes, this is great advice. We now report effect sizes for all our analyses.

#3. Individual Differences
In Study 1, the authors investigated individual differences by using a novel, self-developed scale, the Cognitive Reflection Task of Frederic, and by looking at potential effects of socio-demographic characteristics. On page 12, they state that "none of the individual differences that we measured were meaningfully related to observed levels of consequence neglect (...) Summary statistics describing these null results are shown in

Table 2". While the column labelled "Test Statistic" of Table 2 (p. 13) may suggest that ANOVAs were conducted, the actual type of analysis it is not explicitly stated. This can be easily resolved.
In addition, I was wondering about the analysis regarding the relationship between the number of downward revisions and the Novel Individual Difference Scale – did the authors divide individuals in two groups based on their scores (e.g., low vs. high)? If so, what was the criterium (e.g., median split)? A correlation analysis might also have been appropriate considering that both variables were continuous.

To answer the question, we had originally done Anovas with a median split; we now report that in the manuscript itself. But we agree with you that for some of the analyses with continuous variables, correlation would have been more appropriate, so we now report that instead. It doesn't qualitatively change the results or the conclusions that we draw from them, but it is a better way of doing the analysis, so we are glad to have that included as it strengthens our paper.

#4. Excluded Stimuli
Overall, the authors are very transparent in their reporting. To further increase this transparency, they might consider including one or two examples of solutions that were excluded in the process of stimulus selection for Study 2b (i.e., examples for, "Unclear, confusing, irrelevant and ambiguous solutions were removed. In addition, multi-barreled solutions were removed", p. 18f.).

Great idea. We have included some examples into the paper.

#5. Discussion & Future Directions
In the discussion, the authors mention that "While we found no evidence that demographic differences (including education) moderate the effect of consequence neglect…" (p. 26). I was wondering whether this statement refers to the null effects reported for Study 1, or whether moderating effects of demographics were also tested for the other studies (but not explicitly mentioned). The authors could clarify this by briefly mentioning it in the results sections (or in the discussion).

Yes, that is what we were referring to. We have now clarified that in the text.

In addition to socio-demographics, are there any other potential moderators (and maybe even mediators) the authors can think of that could be addressed in future studies? For example, personal relevance of the subject/domain of the policy under evaluation (or domain expertise), private/professional political engagement (in addition to political orientation, which was investigated in Study 1) may also affect the results. In direct relation to this: Did the authors apply any a-priori exclusion criteria, such as holding political office or pursuing a degree in Political Science or Law? If not, this could also be considered a limitation and could be pursued in a future study (e.g., comparing policymakers to "regular citizens").

Great ideas! We did not explore any of those moderators or apply any of those exclusion criteria. Therefore, we now discuss this in the general discussion as potential future directions.

Furthermore, future studies could also investigate potential effects of external factors such as time pressure or cognitive load, as such factors are often discussed in the context of heuristics and biases.

Ooh, that's a good point. Of course, given that we observe consequence neglect *without* cognitive load or time pressure, we would expect that adding those factors would only exacerbate the problem. We have added a discussion of this issue in the general discussion.

#6. Conclusion
The current conclusion (p. 30) seems to be framed a bit negatively. I think the authors could consider explicitly mentioning the robustness of their findings in order to highlight a very positive aspect of their research.

It is rare for a reviewer to criticize an author for *underselling* the quality of the findings! We were happy to emphasize the robustness of our findings in our discussion, and appreciate the reviewer's recognition and support of the positive aspects of our research. :)

#7. Missing Information in the Appendix / Missing List of Consequences?
On page 12, the authors state that "(A complete list of consequences can be found at OSF at: https://osf.io/8jrew/?view_only=13708524b2ab46889b0eea7bf790a24)".
Unfortunately, this link cannot be accessed directly, as one is directed to a page where one has to log in to OSF and request permission to access the file. In case there is a

typo in the link and it is the same as the one leading to the overall Appendix file: I also checked the current Appendix file I was able to access on OSF (the first link provided in the manuscript), where I was able to find the instructions and self-generated solutions etc., but I did not find the list of consequences in this file.

How embarrassing! This has been rectified.

#8. Inconsistencies in Formatting
There are some inconsistencies in formatting/style in the body of the text (e.g., inconsistent use of leading zeros) and in the reference list (e.g., use of italics for journal names, inconsistencies in capitalization of article titles, abbreviation of first names).
I am uncertain about citation guidelines of the journal, but if APA style is recommended, the authors may also check some deviations from APA in the body of the text (e.g., "Alpha" instead of "α" on p. 12; test statistics are not in italics, e.g., ps and ts).

Thank you for pointing out these errors. We've gone through and fixed the ones you identified, and have done a careful check to make sure there aren't any others.

(Personal comment: I appreciate the insider reference to The Office in the Dunder Mifflin-Scenario :)

:)

Reviewer #2:

I really enjoyed reading your paper and I think you test an interesting and important phenomenon.

Thank you for your kind words, and for appreciating the contribution of our manuscript.

However, there are several major issues that need to be addressed to improve the quality and clarity of your work. Below are my main concerns.
Major issues:
• The paper introduces the term "consequence neglect" but does not explain what it is and how it differs from existing concepts like "focalism in decision making" or "opportunity cost neglect" or "consideration of future consequences". If this is really a new concept, you

need to explain it clearly and discuss its relationship to prior research.

Thank you for pointing this out. We have gone through and defined the terms that we use. We also use formatting to highlight those definitions, putting new concepts in italics when they are introduced and defined so that readers can easily mark that they represent jargon and can understand how we are using them. We paste those definitions below so you don't have to search through the paper for them:

- Thus, we say that someone is exhibiting *consequence neglect* when they fail to consider consequences that materially affect their evaluation of a policy (or a problem-solution more generally), despite being capable of predicting those consequences.
- Other scholarly work related to consequence neglect lies in the phenomenon of *opportunity cost neglect* (Federick et al., 2009), defined as when consumers fail to consider alternative uses for their money when making a consumption decision.
- when people are focused on particular goals, cues, or features, they often neglect other relevant information that would increase the quality of their decision making: a phenomenon known as *focalism*.

• You reference many constructs, such as focalism, opportunity cost neglect, and the illusion of explanatory depth, but the connections between them and your findings are unclear. This creates confusion instead of grounding the study in a solid theoretical framework.

We are sorry that this was unclear as it is quite central to our argument. FOCALISM is a tendency to have tunnel vision - putting most or all of one's attention on one aspect of a decision while neglecting other aspects. CONSEQUENCE NEGLECT is a type of focalism that we have identified, in which people ignore consequences because they are too focused on solving a focal problem. OPPORTUNITY COST NEGLECT is also an example of focalism, specifically in consideration of how one spends money, and could be considered a subset of consequence neglect - opportunity costs are one consequence of a purchase that can be neglected, but there are many other possible consequences that could be neglected as well. Therefore, opportunity costs could fall under the rubric of consequence neglect. We have tried to clarify that in the revisions.

ILLUSION OF EXPLANATORY DEPTH is unrelated to consequence neglect (although some authors have argued that it is an example of focalism). It's only purpose in this paper is that we borrowed our paradigm for studying consequence neglect from the literature on

IOED, so we cited it accordingly. We have clarified that in the most recent version of the manuscript.

• *The Introduction includes fantastic examples (I really loved them!), but they do not align well with what you actually test. The song and the cobra example refer to a different phenomenon (the sequential effects of unintended/ unconsidered/ consequences) than what your studies explore (evaluation shifts after a single iteration of thinking about consequences).*

We're glad to hear you liked our examples. :)

As for your concern about alignment between the examples and what we test, that's because we failed to fully explicate the logic of our studies. We have gone through and elucidated/clarified in the document itself, but we'll summarize it here for your convenience:

Our study measures evaluation shifts after participants are forced to think about consequences of a policy. If they had thought about the negative consequences when they initially had evaluated the policy then we wouldn't see a shift in evaluations - if they had already thought about those consequences at time 1, then there would be no new information provided at time 2 to shift their evaluation. However, forcing them to think about the consequences - both positive and negative - led them to downgrade their evaluation, suggesting that when initially thinking about the policy, they hadn't considered those negative consequences. We argue that this is the proximal mechanism that underlies why the old lady swallows a spider - she was so focused on catching the fly in her stomach, she didn't think about how bad it would be to have a spider in her stomach. Once she thinks about the consequences of spiders in the stomach, she would have shifted her evaluation of the solution to be more negative - reflecting the pattern of data we observe in our participants.

• *How do you actually operationalize consequences? How do you define and distinguish positive vs negative consequences? What exactly do you mean by "material but nonfocal consequences"? How do you decide which consequences are focal and which are not? These definitions need to be explicit and measurable. When you ask participants to generate "positive" and "negative" consequences, it is unclear how these are understood or differentiated. Are they interpreted as "benefits" vs "costs"? How do you ensure consistency across participants? As a result, your studies do not test or control for the instructions given for generating consequences. For example, what happens if participants*

are asked to generate only positive consequences or only negative ones? Or if you frame these as "benefits" and "costs"?

We allow participants to self-operationalize. That is, we tell participants to think about positive and negative consequences, and they interpret that however makes sense to them. So, formally our operationalization of a consequence is “whatever a participant writes down when they are asked to generate a consequence”.

Similarly, we allow participants to determine for themselves what is positive or negative to them. This is important because the same outcome (e.g. reducing immigration) might be viewed as negative to some and positive to others. Participants generate consequences that are idiosyncratically positive and negative for themselves. So, we do not ensure consistency across participants - we view the fact that participants can use their own conception of what positive and negative are as a strength, not a weakness of this paradigm. We assume that if participants only generated what they internally consider to be positive consequences they would evaluate the policy to be stronger, and if they only generated what they internally consider to be negative consequences they would evaluate the policy to be weaker. We actually see that pattern in some pilot tests we ran, but those are on such limited samples (just making sure the paradigm was understood) that we would not put much faith into that finding, nor would we feel comfortable officially reporting those results.

As “material but non-focal consequences” - we define that to be consequences which effect evaluations of the quality of the policy (“material”) but are not directly related to solving the problem that the policy is intended to solve (“non-focal”). That term is never given to participants - it is solely used in the manuscript to describe the focalism hypothesis. We have re-defined it in the manuscript, and hopefully it is clearer now.

• *While you claim that "negative consequences" are central to the phenomenon of consequence neglect (see e.g. your abstract), none of your studies actually examine whether it is specifically the negative (vs positive) consequences that drive the effect. You need to run additional study to test that. Additionally, neither of your studies tests the mechanism behind the “consequence neglect”.*

While it is true that we do not explicitly test whether it is positive vs. negative consequences that drive the effect, it is difficult to think of a pathway by which thinking about positive consequences would lower evaluations of a solution - it should be the case

that identifying more positive elements of a policy would increase evaluations of that policy.   However, that doesn't actually matter for our account - if it happened to be true that generating positive consequences lowered evaluations of a policy, that would still suggest that participants had not considered those consequences when initially generating (Study 2a) or evaluating (Study 2b) the policy.   The reason we have people generate both positive and negative consequences is to give a *more conservative* test of our account. If we were to ask participants to generate consequences without specifying valence, that pragmatically implies that they should generate negative consequences, and that feels like putting our thumb on the scale. (Pilot studies confirm that if you ask for consequences without valence, participants mostly put negative consequences).   That felt heavy handed to us - of course if you ask people to criticize a policy then they will like it less. By forcing them to give positive consequences as well, it prevents pragmatic confounds. But ultimately, from a theoretical perspective, it doesn't actually matter for our account.

• *You mention "boundary conditions" for "consequence neglect" (on page 5), but this discussion is vague. What factors make consequences more or less focal and why? Provide clear predictions and test them*.

This critique is well taken. With that paragraph we were trying to add nuance, but instead we just added confusion. Obviously, there will be boundary conditions for a phenomenon, but without a clear definition of what makes things focal, our treatment of the issue in the initial manuscript just made the theory unfalsifiable. Of course, defining what factors make something focal is well beyond the scope of the present paper - it's the sort of question that makes for a dissertation (or multiple dissertations).

To address this concern, we did a few things. First, we moved the discussion of this issue to the general discussion where we have a little more liberty to be speculative than in the introduction where we're establishing the theory. Second, we explicitly acknowledged that we cannot precisely define when something is focal, and call for future research on the topic. Third, we toned down our claims, removing what we consider the most problematic sentences. In other words, we suggest that there may be boundary conditions, as there undoubtedly are, but rather than making that central, we note it as an important avenue for future exploration.

• *In the Introduction, you suggest that the paper focuses on policy designers, but your studies primarily involve general judgments about various social, business, health-related*

problems, without manipulating individual responsibility for implementing the policies/ solutions. In other words, real-world decision-makers typically focus on one domain (e.g., health, education) and they are incentivized to take responsibility for the outcomes. You did not manipulate this aspect. Instead, you test a wide variety of unrelated problems, which introduces some noise and ignores potential domain effects or the influence of prior expertise. For example, why was alcohol regulation significant in Study 2a but not in later studies? This should be explored. This gap weakens the practical implications of your work. Either tone down the policy implications or run additional studies in which you manipulate and test the effects of domain, context, and expertise.

We had not intended for the paper to focus on policy designers, but rather problem solvers more generally. But upon re-reading the introduction after reading your concern, we can see that you were right - we did imply that. We have gone through and, where appropriate, have changed the word “policy” to “solution” and edited the language more generally to try and make it clear that this is not a phenomenon that we think is specific to policy making. We still believe the effect is germane to policy making - many of our stimuli are policy relevant and policy makers are often engaged in the business of solving problems - but we have reframed the discussion to avoid the impression that this paper is focused on policy specifically, and appreciate that you highlighted that problem with our initial write up.

• *Neither of your study effectively address confounding factors, such as fatigue, participant expertise or fluency (which you admit on pages 27-29). Participants evaluated several diverse problems, which may have impacted their ability to focus.*

Study 3 actually directly addresses fatigue as a factor, but in our previous version of the manuscript we had not incorporated this into our description of the study - that has now been rectified. The “implementation” condition of Study 3 forces participants to evaluate the same number of diverse problems as the “consequences” condition, and serves as a control against fatigue impacting participants’ ability to focus. That has been clarified.

We would argue that participant expertise wasn’t a confounding variable, as participants were randomly assigned to condition, and thus each condition should have contained the same number of experts. This might have increased noise, and may constrain generalization, but it should not have created systematic bias in the data. Moreover, we find no relationship between education, income, and proclivity for consequence neglect. Education and income could be seen as proxies for expertise, albeit admittedly not perfect

ones. So, we see no evidence that expertise confounds the results. However, we have also added to the discussion, explaining that we did not explore participant expertise or test expert populations, and readers should therefore generalize to experts with caution.

As for fluency - as the reviewer notes, we already acknowledged that in the paper. For the reasons we describe in the paper, we do not think it a likely explanation for our results (the 2nd author on this paper is a fluency expert who's bread and butter research is fluency, and even he is skeptical that these are fluency effects). However, it is true that we cannot definitively rule it out, so we do the next best thing, and have expanded our discussion of this issue to make sure readers are aware of it.

• *Your "Individual Difference Scale" (Study 1) lacks validation and it does not work anyway. Given this, along with the lack of control for confounds, I would consider dropping Study 1.*

We agree with the problems in the individual difference scale. In fact, we wouldn't report it at all except for our commitment to open science and reporting all measured variables. It does not really influence any of our conclusions, but since we did run it, we feel that we need to report it. We considered moving Study 1 to an appendix as you suggested, but we ultimately decided against doing so, both because Reviewer 1 asked for additional details there (suggesting that the reviewer wanted the study reported) and because we felt having an additional replication reported added to the robustness of the findings.

• *I may be missing something but why didn't you use repeated measures ANOVA with adjustments for multiple comparisons for your analyses?*

That's a great question. We were interested in the extent that there may be differences in prevalence of consequence neglect over different domains. Throwing all of the data into an ANOVA could have obscured those differences, relative to running separate analyses by domain. As it turns out, the results are so robust by domain that there weren't really differences to have obscured. But we didn't know that when we developed our analysis strategy.

Anyway, given how robust the results were, when one does the ANOVA you asked about, the results are extremely highly significant. The conclusions we would draw would be the same. But we chose to report the multiple t-tests so that readers could get a better sense of just how robust the findings were across domains.

Minor things:

• *Please add an overview of all studies to clarify their purpose and connections.*

Done!

• *Please avoid unnecessary qualifiers like "compelling evidence" or "crucial ways."*

Done!

• *Table 7 needs more details to aid interpretation.*

Done!

In its current form, the paper is very promising but needs some revisions to strengthen the theory, clarify the constructs, and ensure that the findings are robust and relevant. Please rewrite the theory section to clearly position your work in relation to existing research. Consider removing your Study 1 and conducting a new one with better controls for fatigue, expertise, participant understanding of instructions etc. Explore alternative instructions for generating consequences to rule out fluency effects. How does the domain moderate the effects you observe? Consider focusing on testing real decision-makers or manipulating roles to better support your practical implications.

While we did not run a new study, we rewrote the theory section to clarify our position, spent more time elaborating on our approach for dealing with fatigue, strengthened our discussion of expertise and fluency, talked about how robust the findings were across domain (while still acknowledging that our range of tested domains was limited) and discussed issues of generalizing to real policy makers. We tried to be very responsive to the reviewers' concerns, and hope the reviewer agrees that this is a much stronger paper as a result.
